# Structure-aware protein self-supervised learning

**DOI:** 10.1093/bioinformatics/btad189

**Published:** 2023-04-13

**Authors:** Can (Sam) Chen, Jingbo Zhou, Fan Wang, Xue Liu, Dejing Dou

**Affiliations:** School of Computer Science, McGill University, 845 Rue Sherbrooke O, Montreal, Quebec H3A 0G4, Canada; MILA—Quebec AI Institute, 6666 Rue Saint-Urbain, Montreal, Quebec H2S 3H1, Canada; Baidu Research, Xibeiwang East Road, Haidian District, Beijing 100193, China; Baidu Inc., Xuefu Road East, Nanshan District, Shenzhen 518000, China; School of Computer Science, McGill University, 845 Rue Sherbrooke O, Montreal, Quebec H3A 0G4, Canada; BCG X, Level 22, West Tower, Genesis Beijing 8 Xinyuan South Road, Chaoyang District, Beijing 100027, China

## Abstract

**Motivation:**

Protein representation learning methods have shown great potential to many downstream tasks in biological applications. A few recent studies have demonstrated that the self-supervised learning is a promising solution to addressing insufficient labels of proteins, which is a major obstacle to effective protein representation learning. However, existing protein representation learning is usually pretrained on protein sequences without considering the important protein structural information.

**Results:**

In this work, we propose a novel structure-aware protein self-supervised learning method to effectively capture structural information of proteins. In particular, a graph neural network model is pretrained to preserve the protein structural information with self-supervised tasks from a pairwise residue distance perspective and a dihedral angle perspective, respectively. Furthermore, we propose to leverage the available protein language model pretrained on protein sequences to enhance the self-supervised learning. Specifically, we identify the relation between the sequential information in the protein language model and the structural information in the specially designed graph neural network model via a novel pseudo bi-level optimization scheme. We conduct experiments on three downstream tasks: the binary classification into membrane/non-membrane proteins, the location classification into 10 cellular compartments, and the enzyme-catalyzed reaction classification into 384 EC numbers, and these experiments verify the effectiveness of our proposed method.

**Availability and implementation:**

The Alphafold2 database is available in https://alphafold.ebi.ac.uk/. The PDB files are available in https://www.rcsb.org/. The downstream tasks are available in https://github.com/phermosilla/IEConv\_proteins/tree/master/Datasets. The code of the proposed method is available in https://github.com/GGchen1997/STEPS_Bioinformatics.

## 1 Introduction

A variety of machine learning-based biological tasks heavily rely on effective protein representation learning, which aims to extract rich sequential and structural information of a protein into a high-dimensional vector. The learned protein representation can be used in many downstream tasks, such as protein function annotation (Gligorijević et al. 2021), enzyme-catalyzed reaction prediction (Hermosilla et al. 2020), and protein classification (Almagro Armenteros et al. 2017). With this topic attracting lots of research attention recently, different neural network architectures are adopted to learn different levels of protein information based on the labeled protein data via supervised learning. For example, LSTMs (Sønderby et al. 2015) are used to model the sequential information (i.e. primary structure of protein), and variants of graph neural networks (GNNs) (Gligorijević et al. 2021) and convolutional neural networks (Hermosilla et al. 2020) are used to model the structural information. Though these deep learning-based models prove to be effective, one major obstacle to such approach is the lack of labeled data, which is much more severe than the one in computer vision and natural language processing areas since the wet-lab experiment on protein is quite expensive.

Inspired by the remarkable progress of self-supervised learning in other domains, there are a few recent work to perform self-supervised learning for protein from a sequence perspective (Bepler and Berger 2019; Rao et al. 2019, 2020; Vig et al. 2020; Elnaggar et al. 2021; Rives et al. 2021). These sequence-based pretraining methods treat every protein as a sequence of amino acids and use autoregressive or autoencoder methods to obtain the protein representation. Although previous studies (Elnaggar et al. 2021; Rives et al. 2021) found such sequential pretrained protein language models can understand protein structures to some extent, these studies have not explicitly considered modeling structural information of proteins.

Though protein structural information determines a wide range of protein properties (Gligorijević et al. 2021), how to incorporate protein structural information into protein self-supervised learning is overlooked. With the development of structural biology including cryo-EM (Callaway 2020) and Alphafold2 (Jumper et al. 2021), the availability of reliable protein structures is increasing in recent years. Thus, it is desirable to devise a new mechanism to explicitly incorporate protein structural information into self-supervised learning to boost the performance of protein representation learning. Meanwhile, the number of protein sequences is still orders of magnitude larger than the number of proteins with reliable protein structures. Therefore, learning protein representation solely based on the limited number of structural protein data may not be able to show superior performance compared with existing protein language models.

To this end, we propose a novel ‘**ST**rucure-awar**EP**rotein **S**elf-supervised Learning’ (STEPS) method. This method can not only explicitly incorporate protein structural information into protein representations, but also leverage the existing protein language model to enhance protein representation learning. More specifically, we leverage a GNN to model protein structure and propose two novel self-supervised learning tasks to incorporate the distance information and the angle information into protein representation learning. In particular, the GNN model takes the masked protein structure as input and aims to reconstruct the pairwise residue distance information and the dihedral angle information, respectively.

Furthermore, we propose to leverage the available sequential protein language model pretrained on protein sequences (named as protein LM for short) to empower the GNN model via a pseudo bi-level optimization scheme. This optimization scheme aims to effectively transfer the knowledge of the protein LM to the GNN model. The insight is that we identify the relation between the sequential information and the structural information by maximizing the mutual information between the sequential representation and the structural representation. Then a bi-level optimization scheme is devised to exploit the sequential information in the protein LM by leveraging its relation with the structural information in the GNN model. We name this optimization process as ‘pseudo bi-level’ optimization because we update the GNN model in the outer level, but finally keep the parameters of the protein LM fixed in the inner level to avoid distorting the protein LM. Experiments on several downstream tasks verify the effectiveness of STEPS.

In summary, we make the following contributions:

- To the best of our knowledge, we are the first to explicitly incorporate finer protein structural information into self-supervised learning. Two novel self-supervised tasks are proposed to capture the pairwise residue distance information and the dihedral angle information, respectively.
- We adopt a pseudo bi-level optimization scheme to exploit the sequential information in the protein LM.
- We conduct various supervised downstream tasks to verify the effectiveness of STEPS.

## 2 Preliminaries

In this section, we first introduce preliminary concepts and some basic notations used in this article.

**Protein sequence.** Each protein S(V,E) is a sequence of residues linked by peptide bonds. Here, V represents the set of *L* residues in the sequence and E⊆V×V describes the *L—*1 peptide bonds. The protein sequence is mainly composed of 20 different types of amino acids where each unit is commonly named as residue after being joined by peptide bonds.

**Figure 1.**
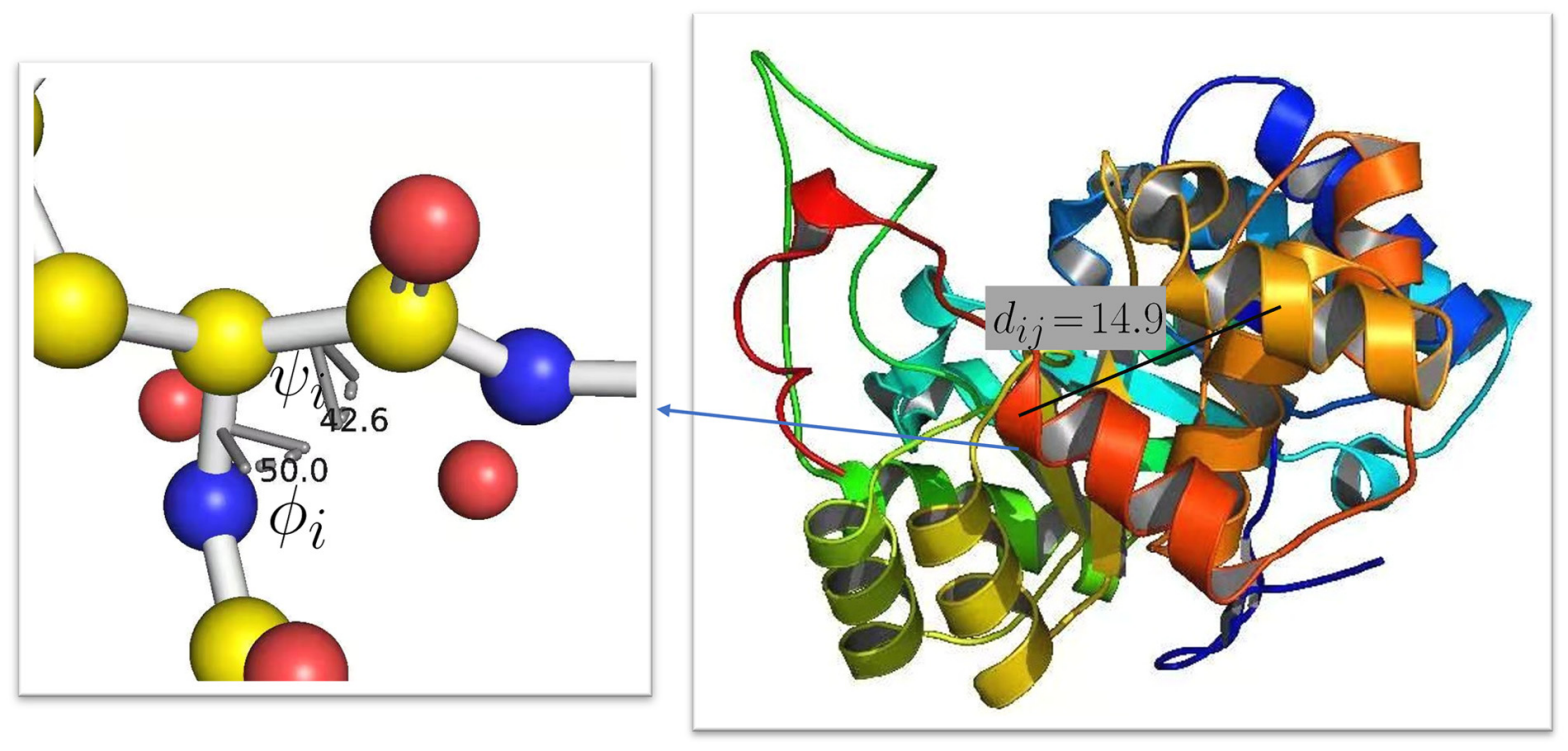
Protein structure.

**Figure 2.**
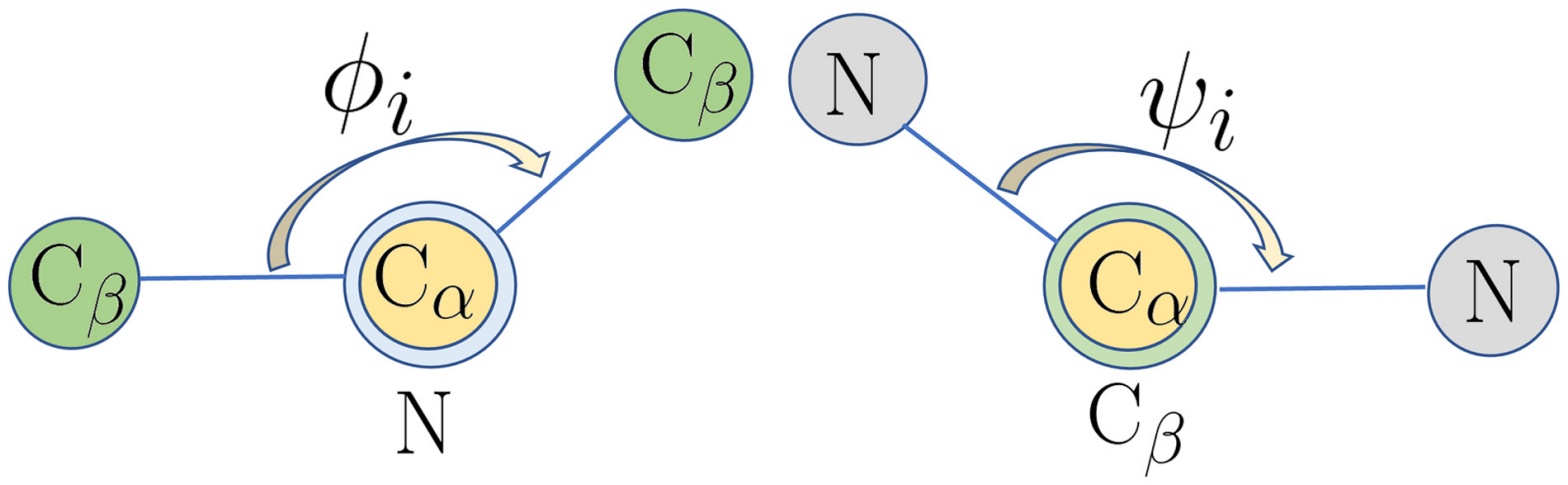
The dihedral angle $\phi_{i}$ and *ψ_i_*.

**Protein structure.** We illustrate an example of protein structure in Fig. 1. As shown in the right part of Fig. 1, pairwise residue distances provide important structural information of the protein. We compute the pairwise residue distance *d_ij_* between residue *i* and residue *j* as the distance between the corresponding $C_{\alpha }$ atoms on the protein backbone. Because of the free rotation of the chemical bonds around alpha carbon, distance information alone cannot fully determine protein backbone structure, which necessitates the dihedral angle information. As shown in the left part of Fig. 1, the protein backbone consists of consecutive units of $C_{\alpha }$–CO–NH, and the rotation information around $C_{\alpha }$ provides further structural information of the protein backbone. A simplified illustration is shown in Fig. 2 where the two dihedral angles $\phi_{i}$ and *ψ_i_* capture the rotation of the N–$C_{\alpha }$ bond and the $C_{\alpha }$–$C_{\beta }$ bond in the residue *i*, respectively. In the protein structure, the dihedral angles $\phi_{i}$ and *ψ_i_* are two important attributes for each residue *i*.

**Protein structure as a graph.** We model each protein as a graph G(V,E), where V denotes the set of nodes in the protein graph and each node represents a residue. Each node v∈V has a node feature $X_{v}$ including the initial residue embedding and the dihedral angle information. There is an edge *e* between two nodes in the graph G if the pairwise residue distance is smaller than a threshold. E represents the set of edges in the protein graph, and $F_{e}$ represents the pairwise residue distance information for e∈E.

## 3 The STEPS framework

**Figure 3.**
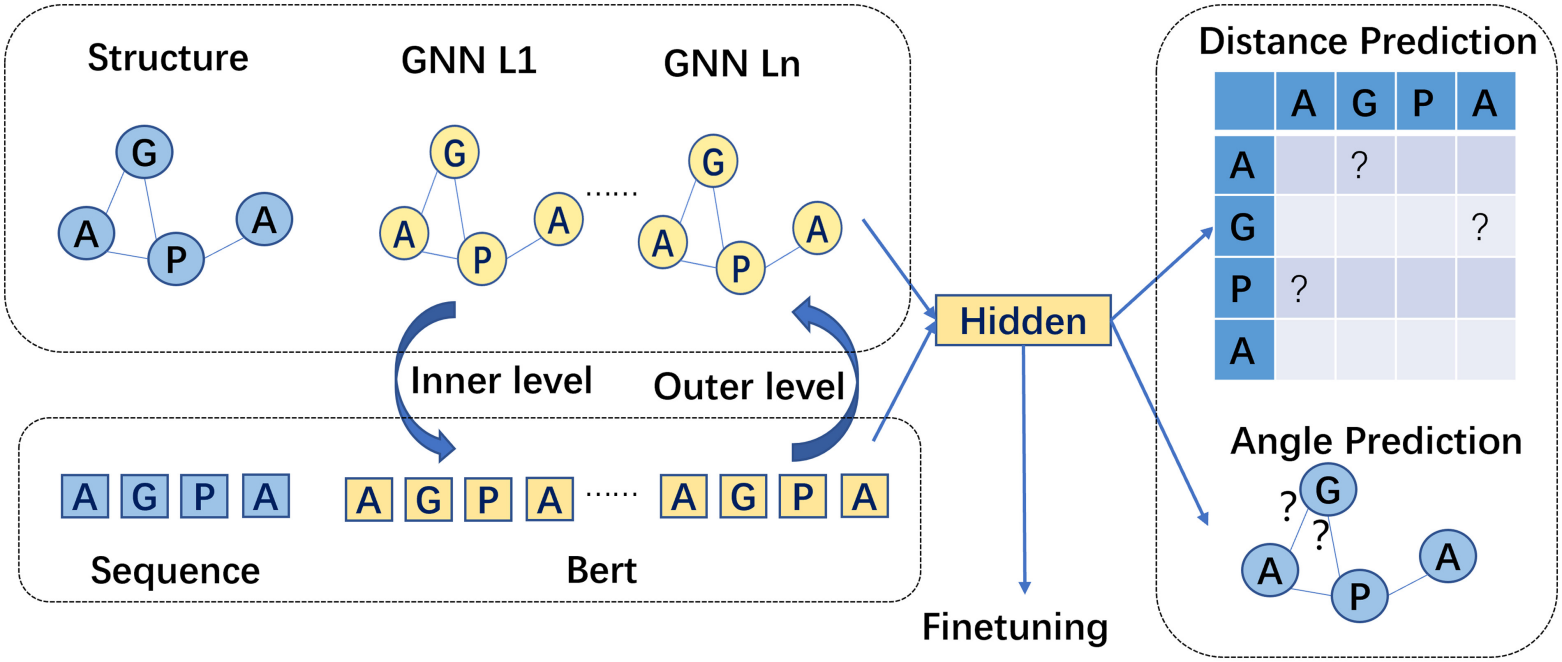
Framework. The GNN model captures protein structural information with two self-supervised tasks: the pairwise distance prediction task and the dihedral angle prediction task. Furthermore, a pseudo bi-level optimization scheme identifies the relation between the protein LM and the GNN model by maximizing the mutual information, which enhances the self-supervised learning.

In this section, we first introduce a protein modeling method using GNN. Second, we present how to use two novel self-supervised tasks to pretrain the GNN model. Finally, we introduce the pseudo bi-level optimization scheme. The overall framework is shown in Fig. 3.

### 3.1 Protein modeling

We model a protein structure as a graph and adopt a GNN model (Xu et al. 2018) to encode the pairwise residue distance information and the dihedral angle information. The designed GNN model parameterized by ω takes as input the protein structural information including node features ***X*** and edge features ***F***, and outputs the node representations and the graph representation.

Denote the node representation for the *i*th node in the *k*th layer of the GNN as $h_{i}^{\left(k\right)}$. The hidden representation $h_{i}^{\left(k\right)}$ is then given by where N(i) denotes the neighbors of node *i* and *e_iv_* denotes the feature of the edge between *i* and *v*. ${\text{AGGREGATE}}^{\left(k\right)}$ is the sum function and ${\text{COMBINE}}^{\left(k\right)}$ is a linear layer for feature transformation following Xu et al. (2018), where we feed the sum of the $h_{i}^{\left(\mathrm{k-1}\right)}$ and $a_{i}^{\left(k\right)}$ as the input for the linear layer. We use the mean READOUT function to output the graph representation of the protein as:


(1) $$a_{i}^{\left(k\right)}={\text{AGGREGATE}}^{\left(k\right)}(e_{iv}h_{v}^{\left(\mathrm{k-1}\right)}||v\in N(i)),$$

(2) $$h_{i}^{\left(k\right)}={\text{COMBINE}}^{\left(k\right)}(h_{i}^{\left(\mathrm{k-1}\right)},a_{i}^{\left(k\right)}),$$

(3) $$h_{G}={\text{MEAN}}^{\left(K\right)}(h_{i}^{\left(K\right)}||i\in V).$$

Note that $h^{0}$ refers to the initial node features X, which mainly include the dihedral angle information and the pretrained node embeddings, which serve as initialization. Specifically, we concatenate the node representation from the pretrained language model and the angle vector into a single input and feed this input into the GNN. The edge feature *e_iv_* refers to the inverse of the square of pairwise residue distance.

To further incorporate the sequential information of a protein, we extract protein sequence representation $h_{i}^{s}$ from the protein LM parameterized by θ, and fuse sequential and structural representation for the residue *i* as: where $h_{i}^{\left(K\right)}$ refers to the final layer hidden representation from the designed GNN model.


(4) $$h_{i}=h_{i}^{s}+h_{i}^{\left(K\right)},$$

### 3.2 Self-supervised learning tasks

We propose two self-supervised learning tasks to explicitly incorporate the distance information and the angle information into protein modeling. The distance prediction task preserves the pairwise residue distance information and the angle prediction task preserves the dihedral angle information. In this way, the GNN model yields protein representation, which well captures the overall protein structural information.

#### 3.2.1 Distance prediction task

The pairwise residue distance determines the overall shape of a protein backbone and thus determines the function of a protein to a large extent. To this end, we introduce a distance prediction task to encode the pairwise residue distance information into the GNN model.

More specifically, we develop a distance prediction network ${\text{NN}}_{\alpha_{\text{dis}}}(\cdot )$, which takes the vector difference between the node hidden representations of residue *i* and residue *j* as input, and aims to predict the pairwise residue distance between *i* and *j*. The intuition for this operation is that the interactions of residues play an important role in determining the diverse functions of protein (Cohen et al. 2009). Therefore, the residues nearby in the protein backbone should have similar representations. Besides, the numerical scale is quite different in the distance matrix even for the same protein. Therefore, it is more effective to formulate this distance prediction task as a multi-class classification problem instead of a regression problem. We divide the distance into *T* uniform bins and every bin corresponds to a certain class. In this way, ${\text{NN}}_{\alpha_{\text{dis}}}(\cdot )$ can be written as: where $d_{ij}^{\prime }\in R^{T}$ represents the predicted pairwise residue distance distribution between the residue *i* and the residue *j* over T classes. We parameterize ${\text{NN}}_{\alpha_{\text{dis}}}(\cdot )$ as two fully-connected layers with a ReLU activation in the middle. For a protein, we optimize the Cross Entropy loss among all residue pairs: where $\text{label}(d_{ij})$ returns the ground truth one-hot label corresponding to the distance *d_ij_*.


(5) $$d_{ij}^{\prime }={\text{NN}}_{\alpha_{\text{dis}}}(h_{i}-h_{j}),$$

(6) $$l_{\mathrm{dis}}=\frac{1}{||V|{|}^{2}}\sum_{i,j}-\text{label}(d_{ij})\;\text{log}(d_{ij}^{\prime }),$$

#### 3.2.2 Angle prediction task

We further propose an angle prediction task for incorporating the dihedral angle information into the GNN model. The angle prediction task aims to predict the dihedral angles of every residue. Due to the free rotation of the chemical bonds around the alpha carbon, dihedral angles of residues are of considerable importance since pairwise residue distances alone cannot determine the protein backbone structure. Note that the dihedral angles are the attributes of each residue of a protein (instead of between two or more residues).

In particular, we propose an angle prediction network ${\text{NN}}_{\alpha_{\text{ang}}}(\cdot )$, which takes the angle-masked residue representation as input and aims to reconstruct the masked angles. For a certain protein, we randomly mask the feature of 15% of residues, and feed the masked protein to the GNN model, which derives the hidden representation of masked residues. Note that the dihedral angles are continuous features and we first normalize the angles into [−1, 1]. After that, we adopt the Radial Basis Function to extend the scalar angle information into an angle feature vector, which serves as input to the GNN model. More specifically, we have where *γ* determines the kernel shape and $\left\{u_{j}\right\}$ represents the center ranging from −1 to 1. Denote the final masked representation of the residue *i* as $h_{i}^{m}$ and then the dihedral angles of the residue *i* can be predicted as: where we parameterize ${\text{NN}}_{\alpha_{\text{ang}}}(\cdot )$ as two fully connected layers with a ReLU activation in the middle. The mean squared error loss is adopted: where M denotes the set of masked residues.


(7) $$E_{k}(x)=\text{exp}(-\gamma ||x-u_{j}|{|}^{2}),$$

(8) $${\bar{\phi }}_{i},{\bar{\psi }}_{i}={\text{NN}}_{\alpha_{\text{ang}}}(h_{i}^{m}),$$

(9) $$l_{\mathrm{angle}}=\sum_{i\in M}{\left(\phi_{i}-{\bar{\phi }}_{i}\right)}^{2}+{\left(\psi_{i}-{\bar{\psi }}_{i}\right)}^{2},$$

To sum up, the loss function for the two self-supervised learning can be compactly written as: where θ, ω, and α denote the parameters of the protein LM, the GNN model, and the prediction networks, respectively.


(10) $$L(\theta ,\omega ,\alpha )=l_{\mathrm{dis}}+l_{\mathrm{angle}},$$

### 3.3 Pseudo bi-level optimization

Yet, directly fusing representations in Equation (4) cannot capture the relation between the sequential information in the protein LM and the structural information in the GNN model. We propose to identify the relation between the protein LM and the GNN model by maximizing the mutual information between the sequential representation and the structural representation. We adopt the Jensen–Shannon MI estimator in Nowozin et al. (2016) to estimate the mutual information. Denote ***x*** as a protein sample from P and $\tilde{x}$ as another protein sample from $\tilde{P}=P$, and then, we have: where $T_{\beta }$ denotes the discriminator parameterized by β and sp is the softplus function. For the details of $T_{\beta }$, we feed the positive and negative examples into a three-layered fully connected network with jumping connections and relu activations, and then output the dotproduct of the two representations. Then, the relation between the sequential parameters θ and the structural parameters ω can be identified by maximizing mutual information:


(11) $$\begin{array}{c} I(\theta ,\omega )=E_{P}[-\text{sp}(-T_{\beta }(h_{\theta }^{s}(x),h_{\omega }^{\left(K\right)}(x))]-\\ E_{P\times \tilde{P}}[\text{sp}(T_{\beta }(h_{\theta }^{s}(x),h_{\omega }^{\left(K\right)}(\tilde{x}))], \end{array}$$

(12) $$\theta^{*}(\omega )=\arg\underset{\theta }{\max}I(\theta ,\omega ).$$

This relation captures the correspondence between the sequential representation and the structural representation for a certain protein (Anfinsen 1973). Note that, we do not actually update θ to θ(ω) in the end, but only leverage the relation to update the GNN model, which means the final adopted θ remains the same as that of the protein LM. In this way, the GNN is updated as: which could better exploit the sequential information in the protein LM.


(13) $$\omega^{\prime }=\arg\underset{\omega }{\min}L(\theta (\omega ),\omega ,\alpha ),$$

This can be formulated as a bi-level optimization problem:


(14) $$\underset{\omega ,\alpha }{\min}\;L(\theta (\omega ),\omega ,\alpha ),$$

(15) $$s.t.\;\theta^{*}(\omega )=\underset{\omega }{\arg\max}I(\theta ,\omega ).$$

Different from the traditional bi-level optimization, we do not update θ in the end similar to Wang et al. (2018), so, we name this scheme as pseudo bi-level optimization. The inner level can be solved approximately by a gradient ascent step:


(16) $$\theta (\omega )=\theta +\eta *\frac{\partial I(\theta ,\omega )}{\partial \theta }.$$

Similarly, the outer level can be solved as:


(17) $$\omega^{\prime }=\omega -\eta^{\prime }*\frac{\partial L(\theta (\omega ),\omega ,\alpha )}{\partial \omega },$$

(18) $$\alpha^{\prime }=\alpha -\eta^{\prime }*\frac{\partial L(\theta (\omega ),\omega ,\alpha )}{\partial \alpha }.$$

## 4 Experiments

In this section, we first introduce the pretraining settings including the datasets and the training details. Second, we evaluate STEPS on three supervised downstream tasks and compare STEPS with existing SOTA methods. At last, we conduct ablation studies to verify the effectiveness of different components in our method STEPS.

### 4.1 Pretraining settings

**Datasets.** We sample an independent set from the alphafold protein database (https://alphafold.ebi.ac.uk/) and remove protein sequences, which have more than 25% sequence similarity with the test proteins, forming a size-40 000 pretraining set.

**Training details.** For the GNN model, we set the dimension of hidden representation as 1280 and the layer number as 2 in our experiments. The threshold to determine whether there is an edge between two residues is set as 7 Å, which is consistent with previous study (Xia and Ku 2021). For ${\text{NN}}_{\alpha_{\text{dis}}}(\cdot )$, we set *T *=* *30 and apply softmax to the output logits. Besides, we adopt the available protein BERT model in Elnaggar et al. (2021) as the pretrained protein language model.

We use the cosine learning rate decay schedule for a total of 10 epochs for pretraining. We set the learning rate for the GNN model as $1e^{-3}$ and the learning rate for the protein LM as $5e^{-5}$ in the pseudo bi-level optimization scheme. The Adam optimizer is adopted to update the GNN parameters with $\beta_{1}=0.9$ and $\beta_{2}=0.999$.

### 4.2 Finetuning

**Downstream tasks.** We finetune the pretrained model on three downstream tasks: the binary classification into membrane/non-membrane proteins, the location classification into 10 cellular compartments (Almagro Armenteros et al. 2017), and the enzyme-catalyzed reaction classification (Hermosilla et al. 2020) into 384 Enzyme Commission numbers. Hereafter, we denote the three tasks as C2, C10, and C384, respectively, for convenience. The train/test sizes of C2, C10, and C384 are 2221/568, 3874/988, and 15 001/2799, respectively. For the binary classification, the membrane/non-membrane protein of the train is 1123/1098 and the membrane/non-membrane protein of the test is 297/271. The performance is evaluated as the mean accuracy (acc) following the setting in Hermosilla et al. (2020).

**Baselines.** We compare STEPS with two groups of baselines: methods without and with pretraining. Methods without pretraining include:

- Blast (Radivojac et al. 2013): a sequence in the test set receives labels from all labeled sequences in the training set and the prediction is obtained as the highest one. Similar to Gligorijević et al. (2021), we remove all training sequences with an *E*-value threshold 1e-3 to prevent label transfer from homologous sequences.
- IEConv (Hermosilla et al. 2020): it introduces a novel convolution operator and hierarchical pooling operators to model different particularities for a protein.

Methods with pretraining are:

- Pre-LM (Elnaggar et al. 2021): it adopts the protein BERT model pretrained on Uniref100 and adds a fully connected layer with tanh activation as the head for finetuning. The head takes the mean pooling over residue representations as input and outputs scores.
- DeepFRI (Gligorijević et al. 2021): this method adopts the Graph Convolutional Network (GCN) to predict protein functions by leveraging structural features. It also adopts a pretrained language model to obtain the residue embedding as the input of the GCN model.
- STEPS-w/oLM: we pretrain the GNN model in STEPS without considering protein LMs. We add a layer on GNN for finetuning. The initial node representation for GNN is the concatenation of the one-hot encoding of amino acid and the dihedral angle vectors.

For the proposed STEPS, we finetune both the GNN model and the linear head. Besides, we use STEPS-H to denote the STEPS with only finetuning the linear head.

**Training details.** For all methods and all datasets, we adopt a cosine learning rate decay with an initial learning rate 1e-4 and train the models for five epochs with the Adam optimizer for a fair comparison.

### 4.3 Result analysis

As shown in Tables 1–3, we report the best results in bold and mark the second best results (excluding STEPS-H) among two groups of baselines by underlines. First, we can observe that STEPS has consistent gains over all comparison methods in the three downstream tasks. More specifically, compared with the second best results, STEPS achieves 1.39% relative gain in C2, 2.63% relative gain in C10, and 36.68% performance gain in C384, which proves the effectiveness of STEPS. Note that STEPS performs better than STEPS-H, which means further finetuning the GNN model on a specific task yields better representation. It is worth noting that STEPS significantly outperforms its baselines in C384. A potential reason is that protein structure primarily determines the specific binding sites of an enzyme. Therefore, as the first method to incorporate the structural information into protein pretraining, STEPS performs much better than other methods on the enzyme-catalyzed reaction classification task (i.e. C384). We conduct additional experiments where the test set structures are from Alphafold instead of the PDB database on C384, and the results are very similar. The STEPS acc on C384 is 65.38% when using the structures from Alphafold and is 65.74% when using the PDB database. Furthermore, we can observe that Pre-LM and STEPS-w/oLM perform worse than STEPS, which verifies the necessity of structural information and sequential information for protein pretraining. At last, we can observe STEPS-w/oLM performs better than Pre-LM by 19.82% in C2, 7.51% in C10, and 2.57% in C384, which indicates structural information is more important than sequential information for protein representation learning.

**Table 1.** Experimental results on C2 for comparison.

Method	Blast	IEConv	DeepFRI	Pre-LM	STEPS-w/oLM	STEPS-H	STEPS
Acc (%)	65.14	62.15	88.38	58.00	77.82	87.68	**89.61**

**Table 2.** Experimental results on C10 for comparison.

Method	Blast	IEConv	DeepFRI	Pre-LM	STEPS-w/oLM	STEPS-H	STEPS
Acc (%)	31.78	30.99	69.23	35.00	42.51	69.84	**71.05**

**Table 3.** Experimental results on C384 for comparison.

Method	Blast	IEConv	DeepFRI	Pre-LM	STEPS-w/oLM	STEPS-H	STEPS
Acc (%)	12.83	28.70	15.72	1.32	3.89	50.13	**65.38**

### 4.4 Ablation studies

In this section, we conduct ablation studies to verify the effectiveness of different components in STEPS.

#### 4.4.1 Mutual information

**Figure 4.**
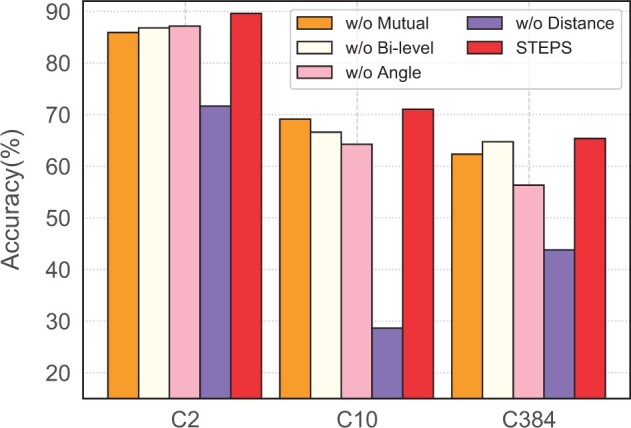
Ablation studies.

We first remove the mutual information from STEPS, denoted as w/o Mutual, and only use the self-supervised learning losses. As shown in Fig. 4, this removal leads to decreases on all three tasks, which demonstrates the importance of the mutual information.

#### 4.4.2 Pseduo bi-level optimization

To verify the effectiveness of the pseudo bi-level optimization, we remove this part from STEPS and only optimize the GNN model, which is denoted as w/o Bi-level. Besides, we consider alternate optimization and joint optimization between the protein LM and the GNN model. We find alternate optimization and joint optimization do not perform well and are even much worse than without these optimizations, so we do not report these results here. The reason may be the protein LM collapse during updating (Dodge et al. 2020). As shown in Fig. 4, STEPS consistently outperforms STEPS w/o Bi-level in all tasks, which verifies the effectiveness of the proposed pseduo bi-level optimization.

#### 4.4.3 Self-supervised learning tasks

We then demonstrate the effectiveness of the two self-supervised learning tasks: the pairwise residue distance prediction task and the dihedral angle prediction task. We remove the angle prediction task from STEPS and denote it as w/o Angle. Similarly, we remove the distance prediction task from STEPS and denote it as w/o Distance. As shown in Fig. 4, removing either task leads to noticeable performance degradation, which proves the necessity of both self-supervised learning tasks. Moreover, we observe that STEPS w/o Distance results in 15.50% performance decline in C2, 35.63% performance decline in C10, and 12.54% performance decline in C384 compared with STEPS w/o Angle. This phenomenon indicates that the pairwise residue distance information plays a more important role than the dihedral angle information in protein modeling. We follow the work (Xia and Ku 2021; Fang et al. 2022) to model the distance prediction as a multi-class classification problem rather than a regression problem. The regression formulation leads to 3.87% performance decline in C2, 1.01% performance decline in C10, and 2.11% performance decline in C384.

## 5 Related work

### 5.1 Protein representation learning

Protein representation learning methods are mainly classified into two categories: sequence-based methods and structure-based methods. Sequence-based methods model a protein via its 1D amino acid sequence. For example, Hou et al. (2018) adopt 1D convolutional neural networks to derive hidden representation for classification. Structure-based methods consider the 3D structure of proteins. For example, Townshend et al. (2019) leverage 3D convolutional neural networks for protein quality assessment and protein contact prediction. Hermosilla et al. (2020) propose novel convolutional operators and pooling operators to model the primary, secondary, and tertiary structure effectively, which demonstrates strong performance on protein function prediction tasks. Gligorijević et al. (2021) leverage the LSTM model to encode the protein sequence and the GCN model to encode the protein tertiary structure for function prediction. Somnath et al. (2021) connect protein surface to structure modeling and sequence modeling where the learned representation achieves good performance on several downstream tasks.

### 5.2 Protein pretraining

There are a few studies to perform pretraining on protein sequences (Bepler and Berger 2019; Wang et al. 2023; Zhou et al. 2023). Bepler and Berger (2019) propose to train an LSTM on protein sequences, which could implicitly incorporate structural information from the global structural similarity between proteins and the contact maps for individual proteins, while STEPS uses novel self-supervised tasks to explicitly model protein structure. Our distance prediction task and the contact prediction task in Bepler and Berger (2019, 2021) can both incorporate the distance information into the learned protein representation. The difference is that the distance prediction task models the distance as a multi-class classification and this can explicitly consider more protein structural information compared with the binary classification of the contact prediction. Rives et al. (2021) are the first to model protein sequences with self-attention, and the learned representation of the pretrained language model contains the protein information of structure and function. Elnaggar et al. (2021) try to train autoregressive language models and autoencoder models on large datasets, and validate the feasibility of training big language models on proteins. Rao et al. (2020) and Vig et al. (2020) study the transformer attention maps from the unsupervised learned language model and uncover the relationship between the attention map and the protein contact map. Fang et al. (2022) design similar self-supervised learning tasks for molecules while STEPS considers different information including the pairwise residue distance information and the dihedral angle information for protein modeling. Besides, STEPS models protein from both sequence and structure views while Fang et al. (2022) model molecule from only the structure view. A concurrent work of STEPS (Zhang et al. 2022, 2023) adopts a well-designed GNN for protein pretraining. A future direction may be adopting the pretrained protein models for protein optimization (Chen et al. 2023).

### 5.3 Bi-level optimization

Bi-level optimization is a special kind of optimization problem where one level of problem is embedded in the other level. Bi-level optimization has been widely used in the deep learning community due the hierarchy problem structure in many applications (Hospedales et al. 2020; Chen et al. 2021, 2022a,b,c) including neural architecture search, instance weighting, initial condition, learning to optimize, data augmentation, etc. In this article, similar to Wang et al. (2018), we develop a pseudo bi-level optimization scheme to identify the relation between the sequential information in the protein LM and the structural information in the GNN model, which can help exploit the sequential information in the protein LM.

## 6 Conclusion

In this article, to effectively capture protein structural information, we investigate a novel structure-aware self-supervised protein learning approach. Along this line, two novel self-supervised learning tasks on a GNN model are adopted to capture the pairwise residue distance information and the dihedral angle information, respectively. Also, to leverage the pretrained sequential protein language model to further improve the representation learning, we propose a pseudo bi-level optimization scheme to transfer the knowledge of the protein LM to the GNN model. Finally, the experimental results on several benchmarks for protein classification show the effectiveness and the generalizability of our method STEPS.
